# (*E*)-1-[(2,4,6-Tri­bromo­phen­yl)diazen­yl]naphthalen-2-ol

**DOI:** 10.1107/S1600536813018977

**Published:** 2013-07-13

**Authors:** Souheyla Chetioui, Issam Boudraa, Sofiane Bouacida, Abdelkader Bouchoul, Salah Eddine Bouaoud

**Affiliations:** a Unité de Recherche de Chimie de l’Environnement et Moléculaire Structurale (CHEMS), Faculté des Sciences Exactes, Département de Chimie, Université Constantine 1, 25000 Constantine, Algeria

## Abstract

The title azo mol­ecule, C_16_H_9_Br_3_N_2_O, adopts a *trans* conformation with respect to the azo N=N double bond. An intra­molecular O—H⋯N hydrogen bond forms an *S*(6) ring motif. The dihedral angle between the naphthalene ring system and the benzene ring is 33.80 (16)°. In the crystal, mol­ecules are stacked in columns along the *a* axis by π–π inter­actions [centroid–centroid distances = 3.815 (3) and 3.990 (3) Å].

## Related literature

For applications of azo compounds, see: Gale *et al.* (1998 ▶). For the synthesis of similar compounds, see: Wang *et al.* (2003 ▶); Heinrich *et al.* (2007 ▶). For bond lengths and angles in related azo compounds, see: Deveci *et al.* (2005 ▶); El-Ghamry *et al.* (2008 ▶).
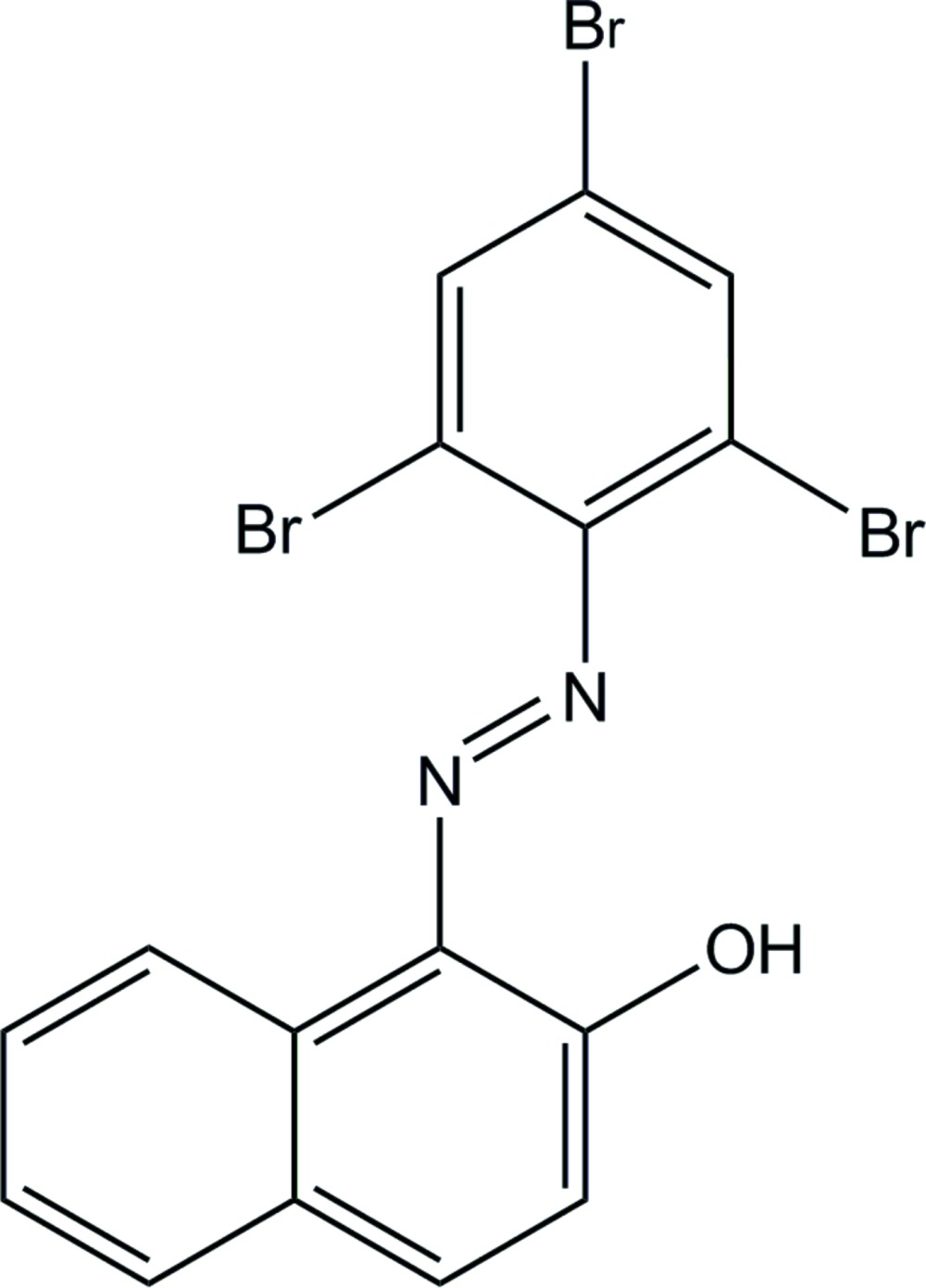


## Experimental

### 

#### Crystal data

C_16_H_9_Br_3_N_2_O
*M* *_r_* = 484.98
Orthorhombic, 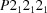

*a* = 3.9904 (11) Å
*b* = 15.689 (4) Å
*c* = 24.580 (7) Å
*V* = 1538.8 (7) Å^3^
*Z* = 4
Mo *K*α radiation
μ = 7.87 mm^−1^
*T* = 293 K
0.03 × 0.02 × 0.02 mm

#### Data collection

Bruker APEXII CCD diffractometer
Absorption correction: multi-scan (*SADABS*; Sheldrick, 2004 ▶) *T*_min_ = 0.244, *T*_max_ = 0.332
13143 measured reflections
3841 independent reflections
2910 reflections with *I* > 2σ(*I*)
*R*_int_ = 0.046

#### Refinement

*R*[*F* ^2^ > 2σ(*F* ^2^)] = 0.034
*wR*(*F* ^2^) = 0.066
*S* = 0.96
3841 reflections
199 parameters
H-atom parameters constrained
Δρ_max_ = 0.44 e Å^−3^
Δρ_min_ = −0.44 e Å^−3^
Absolute structure: Flack (1983 ▶), 1553 Friedel pairs
Flack parameter: 0.004 (13)

### 

Data collection: *APEX2* (Bruker, 2006 ▶); cell refinement: *SAINT* (Bruker, 2006 ▶); data reduction: *SAINT*; program(s) used to solve structure: *SHELXS97* (Sheldrick, 2008 ▶); program(s) used to refine structure: *SHELXL97* (Sheldrick, 2008 ▶); molecular graphics: *ORTEP-3 for Windows* (Farrugia, 2012 ▶); software used to prepare material for publication: *WinGX* (Farrugia, 2012 ▶).

## Supplementary Material

Crystal structure: contains datablock(s) global, I. DOI: 10.1107/S1600536813018977/is5290sup1.cif

Structure factors: contains datablock(s) I. DOI: 10.1107/S1600536813018977/is5290Isup2.hkl

Supplementary material file. DOI: 10.1107/S1600536813018977/is5290Isup3.cml

Additional supplementary materials:  crystallographic information; 3D view; checkCIF report

## Figures and Tables

**Table 1:** Hydrogen-bond geometry (Å, °)

*D*—H⋯*A*	*D*—H	H⋯*A*	*D*⋯*A*	*D*—H⋯*A*
O1—H1⋯N1	0.82	1.85	2.561 (4)	144

**Fig. 1.**
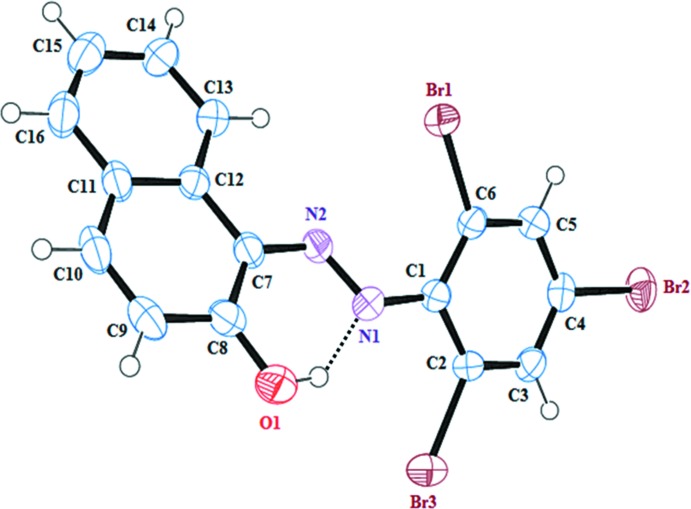
The molecular structure of the title compound, with atom labels and 50% probability displacement ellipsoids for non-H atoms. The dashed line indicates the O—H···N hydrogen bond.

**Fig. 2.**
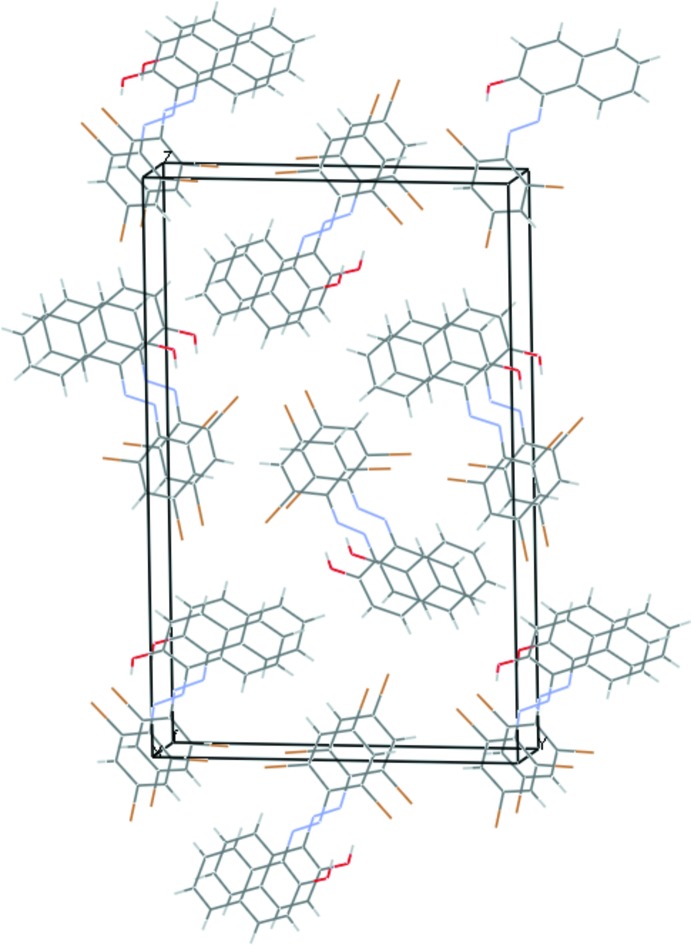
A packing diagram of the title compound viewed along the a axis.

## supplementary crystallographic information

### Comment

It has been known for many years that the azo compounds are a widely used class of dyes due to their application in various fields such as the dyeing of textile fibers, the coloring of different materials, colored plastics and electrochemical sensors (Gale *et al.*, 1998). Azo dyes are synthetic colours that contain an azo group, as part of the structure. They are characterized by the azo linkage (–N=N–). Azo groups do not occur naturally. Many azo compounds have been synthesized by the diazotization and diazo coupling reaction (Wang *et al.*, 2003), which entails an electrophilic substitution reaction where an aryl diazonium-cation attacks another aryl ring, since diazonium salts are often unstable near room temperature; the azo coupling reactions are typically conducted near ice temperature.

The pH of solution is quite important; it must be mildly acidic or neutral, since no reaction takes place if the pH is too low (Heinrich *et al.*, 2007). We report herein the crystal structure of the title compound (Fig. 1), obtained through the diazotization of 2,4,6-tribromoaniline followed by a coupling reaction with 2-naphthol. In the molecule of the title compound, all bond lengths are in good agreement with those reported for other azo compounds (Deveci *et al.*, 2005; El-Ghamry *et al.*, 2008). The bond lengths and angles are within normal ranges. The naphthalene ring system is oriented at a dihedral angle of 33.80 (16)° with respect to the benzene ring. In the crystal, molecules are packed into columns along the *a* axis by π–π interactions between adjacent molecules with the closest approach between centroids of aromatic rings being 3.815 (3) Å (Fig. 2).

### Experimental

The title compound was prepared by the previously reported method in the literature; following the classical method of synthesis of other aromatic azo-compounds, diazotization of 2,4,6-tribromoaniline followed by a coupling reaction with 2-naphthol (Wang *et al.*, 2003). This gives a red powder which was crystallized from methanol solution leading to red prismatic crystals.

### Refinement

All H atoms have been placed in geometrically idealized positions (C—H = 0.93 Å and O—H = 0.82 Å) and refined as riding with *U*_iso_(H) = 1.2*U*_eq_(C) or 1.5*U*_eq_(O).

### Crystal data

**Table:**

C_16_H_9_Br_3_N_2_O	*D*_x_ = 2.093 Mg m^−^^3^
*M**_r_* = 484.98	Melting point: 422 K
Orthorhombic, *P*2_1_2_1_2_1_	Mo *K*α radiation, λ = 0.71073 Å
Hall symbol: P 2ac 2ab	Cell parameters from 2052 reflections
*a* = 3.9904 (11) Å	θ = 3.1–28.6°
*b* = 15.689 (4) Å	µ = 7.87 mm^−^^1^
*c* = 24.580 (7) Å	*T* = 293 K
*V* = 1538.8 (7) Å^3^	Prism, red
*Z* = 4	0.03 × 0.02 × 0.02 mm
*F*(000) = 928	

### Data collection

**Table:**

Bruker APEXII CCD diffractometer	3841 independent reflections
Radiation source: fine-focus sealed tube	2910 reflections with *I* > 2σ(*I*)
Graphite monochromator	*R*_int_ = 0.046
φ and ω scans	θ_max_ = 28.3°, θ_min_ = 2.1°
Absorption correction: multi-scan (*SADABS*; Sheldrick, 2004)	*h* = −5→5
*T*_min_ = 0.244, *T*_max_ = 0.332	*k* = −20→20
13143 measured reflections	*l* = −32→32

### Refinement

**Table:**

Refinement on *F*^2^	Secondary atom site location: difference Fourier map
Least-squares matrix: full	Hydrogen site location: inferred from neighbouring sites
*R*[*F*^2^ > 2σ(*F*^2^)] = 0.034	H-atom parameters constrained
*wR*(*F*^2^) = 0.066	*w* = 1/[σ^2^(*F*_o_^2^) + (0.0241*P*)^2^] where *P* = (*F*_o_^2^ + 2*F*_c_^2^)/3
*S* = 0.96	(Δ/σ)_max_ < 0.001
3841 reflections	Δρ_max_ = 0.44 e Å^−^^3^
199 parameters	Δρ_min_ = −0.44 e Å^−^^3^
0 restraints	Absolute structure: Flack (1983), 1553 Friedel pairs
Primary atom site location: structure-invariant direct methods	Flack parameter: 0.004 (13)

### Special details

**Table:**

Geometry. All e.s.d.'s (except the e.s.d. in the dihedral angle between two l.s. planes) are estimated using the full covariance matrix. The cell e.s.d.'s are taken into account individually in the estimation of e.s.d.'s in distances, angles and torsion angles; correlations between e.s.d.'s in cell parameters are only used when they are defined by crystal symmetry. An approximate (isotropic) treatment of cell e.s.d.'s is used for estimating e.s.d.'s involving l.s. planes.
Refinement. Refinement of *F*^2^ against ALL reflections. The weighted *R*-factor *wR* and goodness of fit *S* are based on *F*^2^, conventional *R*-factors *R* are based on *F*, with *F* set to zero for negative *F*^2^. The threshold expression of *F*^2^ > σ(*F*^2^) is used only for calculating *R*-factors(gt) *etc*. and is not relevant to the choice of reflections for refinement. *R*-factors based on *F*^2^ are statistically about twice as large as those based on *F*, and *R*- factors based on ALL data will be even larger.

### Fractional atomic coordinates and isotropic or equivalent isotropic displacement parameters (Å^2^)

**Table:**

	*x*	*y*	*z*	*U*_iso_*/*U*_eq_	
Br1	−0.12633 (11)	−0.15833 (2)	0.00161 (2)	0.0379 (1)	
Br2	0.51026 (12)	0.09227 (3)	0.12476 (2)	0.0481 (2)	
Br3	0.12463 (15)	0.17248 (3)	−0.08940 (2)	0.0557 (2)	
O1	−0.4121 (9)	0.02557 (17)	−0.18018 (11)	0.0512 (10)	
N1	−0.0964 (9)	−0.01183 (18)	−0.09266 (12)	0.0352 (10)	
N2	−0.0368 (8)	−0.08820 (18)	−0.10973 (11)	0.0317 (10)	
C1	0.0433 (10)	0.0081 (2)	−0.04134 (14)	0.0304 (11)	
C2	0.0607 (9)	−0.0479 (2)	0.00323 (14)	0.0304 (11)	
C3	0.2004 (11)	−0.0226 (2)	0.05163 (14)	0.0333 (12)	
C4	0.3199 (11)	0.0592 (2)	0.05766 (15)	0.0342 (11)	
C5	0.2983 (11)	0.1171 (2)	0.01563 (15)	0.0373 (14)	
C6	0.1572 (11)	0.0906 (2)	−0.03316 (14)	0.0337 (11)	
C7	−0.1725 (10)	−0.1097 (2)	−0.15902 (14)	0.0307 (11)	
C8	−0.3485 (12)	−0.0537 (3)	−0.19353 (15)	0.0387 (14)	
C9	−0.4639 (11)	−0.0836 (3)	−0.24475 (15)	0.0432 (15)	
C10	−0.4111 (11)	−0.1653 (3)	−0.25976 (14)	0.0437 (15)	
C11	−0.2321 (11)	−0.2247 (3)	−0.22725 (14)	0.0355 (11)	
C12	−0.1079 (10)	−0.1969 (2)	−0.17622 (14)	0.0299 (11)	
C13	0.0637 (11)	−0.2558 (2)	−0.14376 (15)	0.0360 (14)	
C14	0.1125 (12)	−0.3377 (2)	−0.16128 (16)	0.0443 (14)	
C15	−0.0059 (13)	−0.3643 (3)	−0.21170 (17)	0.0487 (16)	
C16	−0.1756 (13)	−0.3094 (3)	−0.24383 (16)	0.0470 (16)	
H1	−0.33456	0.03546	−0.14993	0.0768*	
H3	0.21435	−0.06078	0.08045	0.0402*	
H5	0.37633	0.17259	0.01985	0.0449*	
H9	−0.57652	−0.04673	−0.26807	0.0520*	
H10	−0.49596	−0.18353	−0.29301	0.0524*	
H13	0.14544	−0.23913	−0.10996	0.0432*	
H14	0.22647	−0.37609	−0.13916	0.0531*	
H15	0.03174	−0.41991	−0.22331	0.0588*	
H16	−0.25621	−0.32774	−0.27734	0.0563*	

### Atomic displacement parameters (Å^2^)

**Table:**

	*U*^11^	*U*^22^	*U*^33^	*U*^12^	*U*^13^	*U*^23^
Br1	0.0446 (2)	0.0286 (2)	0.0405 (2)	−0.0047 (2)	0.0045 (2)	0.0003 (2)
Br2	0.0505 (3)	0.0582 (3)	0.0356 (2)	−0.0074 (2)	−0.0023 (2)	−0.0136 (2)
Br3	0.0867 (4)	0.0324 (2)	0.0481 (2)	−0.0006 (3)	0.0002 (3)	0.0081 (2)
O1	0.072 (2)	0.0408 (16)	0.0409 (16)	0.0086 (18)	−0.0086 (17)	0.0069 (13)
N1	0.045 (2)	0.0281 (16)	0.0324 (16)	−0.0006 (16)	0.0006 (17)	−0.0034 (13)
N2	0.035 (2)	0.0328 (16)	0.0273 (14)	−0.0046 (16)	0.0039 (14)	−0.0007 (13)
C1	0.031 (2)	0.0277 (18)	0.0324 (19)	0.0023 (18)	0.0052 (18)	−0.0024 (15)
C2	0.031 (2)	0.0263 (17)	0.0339 (18)	0.0023 (16)	0.0058 (19)	−0.0031 (16)
C3	0.036 (2)	0.033 (2)	0.031 (2)	0.0011 (19)	0.0048 (18)	0.0007 (16)
C4	0.031 (2)	0.041 (2)	0.0306 (19)	0.000 (2)	0.0028 (19)	−0.0106 (17)
C5	0.047 (3)	0.028 (2)	0.037 (2)	−0.0053 (19)	0.0080 (19)	−0.0072 (16)
C6	0.042 (2)	0.0272 (18)	0.0318 (19)	0.001 (2)	0.0068 (19)	0.0040 (16)
C7	0.033 (2)	0.035 (2)	0.0241 (18)	−0.0030 (19)	0.0019 (17)	0.0007 (16)
C8	0.045 (3)	0.036 (2)	0.035 (2)	−0.002 (2)	0.001 (2)	0.0053 (17)
C9	0.044 (3)	0.056 (3)	0.0297 (19)	−0.002 (2)	−0.005 (2)	0.0108 (19)
C10	0.048 (3)	0.059 (3)	0.0240 (18)	−0.013 (3)	0.0018 (18)	−0.0022 (19)
C11	0.037 (2)	0.045 (2)	0.0245 (19)	−0.011 (2)	0.0032 (18)	−0.0023 (18)
C12	0.028 (2)	0.0331 (19)	0.0286 (18)	−0.0085 (19)	0.0068 (17)	−0.0003 (15)
C13	0.040 (3)	0.038 (2)	0.0301 (19)	−0.002 (2)	0.0008 (18)	−0.0044 (16)
C14	0.052 (3)	0.036 (2)	0.045 (2)	0.005 (2)	0.006 (2)	−0.0030 (19)
C15	0.048 (3)	0.041 (2)	0.057 (3)	−0.004 (2)	0.013 (3)	−0.015 (2)
C16	0.054 (3)	0.053 (3)	0.034 (2)	−0.014 (2)	0.006 (2)	−0.016 (2)

### Geometric parameters (Å, º)

**Table:**

Br1—C2	1.887 (3)	C9—C10	1.350 (7)
Br2—C4	1.889 (4)	C10—C11	1.420 (6)
Br3—C6	1.892 (3)	C11—C16	1.408 (7)
O1—C8	1.311 (5)	C11—C12	1.418 (5)
O1—H1	0.8200	C12—C13	1.400 (5)
N1—N2	1.292 (4)	C13—C14	1.369 (5)
N1—C1	1.414 (5)	C14—C15	1.390 (6)
N2—C7	1.369 (5)	C15—C16	1.351 (7)
C1—C2	1.406 (5)	C3—H3	0.9300
C1—C6	1.387 (5)	C5—H5	0.9300
C2—C3	1.373 (5)	C9—H9	0.9300
C3—C4	1.377 (5)	C10—H10	0.9300
C4—C5	1.378 (5)	C13—H13	0.9300
C5—C6	1.389 (5)	C14—H14	0.9300
C7—C8	1.409 (6)	C15—H15	0.9300
C7—C12	1.455 (5)	C16—H16	0.9300
C8—C9	1.420 (6)		
			
C8—O1—H1	109.00	C12—C11—C16	119.4 (4)
N2—N1—C1	115.0 (3)	C10—C11—C12	118.2 (4)
N1—N2—C7	116.3 (3)	C7—C12—C13	122.8 (3)
N1—C1—C2	125.2 (3)	C11—C12—C13	118.2 (3)
C2—C1—C6	117.0 (3)	C7—C12—C11	119.0 (3)
N1—C1—C6	117.7 (3)	C12—C13—C14	120.7 (3)
Br1—C2—C3	116.4 (2)	C13—C14—C15	120.9 (4)
C1—C2—C3	121.0 (3)	C14—C15—C16	120.0 (4)
Br1—C2—C1	122.5 (3)	C11—C16—C15	120.9 (4)
C2—C3—C4	120.2 (3)	C2—C3—H3	120.00
Br2—C4—C5	119.9 (3)	C4—C3—H3	120.00
C3—C4—C5	120.8 (3)	C4—C5—H5	121.00
Br2—C4—C3	119.3 (3)	C6—C5—H5	121.00
C4—C5—C6	118.4 (3)	C8—C9—H9	120.00
Br3—C6—C5	117.1 (2)	C10—C9—H9	120.00
C1—C6—C5	122.5 (3)	C9—C10—H10	118.00
Br3—C6—C1	120.4 (3)	C11—C10—H10	118.00
N2—C7—C12	114.8 (3)	C12—C13—H13	120.00
C8—C7—C12	120.0 (3)	C14—C13—H13	120.00
N2—C7—C8	125.2 (3)	C13—C14—H14	120.00
O1—C8—C9	118.2 (4)	C15—C14—H14	120.00
C7—C8—C9	119.3 (4)	C14—C15—H15	120.00
O1—C8—C7	122.5 (3)	C16—C15—H15	120.00
C8—C9—C10	120.3 (4)	C11—C16—H16	120.00
C9—C10—C11	123.2 (4)	C15—C16—H16	120.00
C10—C11—C16	122.5 (4)		
			
C1—N1—N2—C7	179.2 (3)	C12—C7—C8—O1	−179.7 (4)
N2—N1—C1—C2	−38.6 (5)	C12—C7—C8—C9	0.6 (6)
N2—N1—C1—C6	144.9 (4)	N2—C7—C12—C11	−178.7 (3)
N1—N2—C7—C8	4.8 (6)	N2—C7—C12—C13	3.3 (6)
N1—N2—C7—C12	−178.4 (3)	C8—C7—C12—C11	−1.6 (6)
N1—C1—C2—Br1	−3.5 (5)	C8—C7—C12—C13	−179.6 (4)
N1—C1—C2—C3	−179.8 (4)	O1—C8—C9—C10	−178.5 (4)
C6—C1—C2—Br1	173.1 (3)	C7—C8—C9—C10	1.3 (7)
C6—C1—C2—C3	−3.2 (6)	C8—C9—C10—C11	−2.1 (7)
N1—C1—C6—Br3	−0.1 (5)	C9—C10—C11—C12	1.0 (6)
N1—C1—C6—C5	179.9 (4)	C9—C10—C11—C16	−179.2 (4)
C2—C1—C6—Br3	−176.9 (3)	C10—C11—C12—C7	0.9 (6)
C2—C1—C6—C5	3.1 (6)	C10—C11—C12—C13	179.0 (4)
Br1—C2—C3—C4	−175.1 (3)	C16—C11—C12—C7	−179.0 (4)
C1—C2—C3—C4	1.4 (6)	C16—C11—C12—C13	−0.9 (6)
C2—C3—C4—Br2	179.9 (3)	C10—C11—C16—C15	−179.6 (4)
C2—C3—C4—C5	0.7 (6)	C12—C11—C16—C15	0.2 (7)
Br2—C4—C5—C6	179.9 (3)	C7—C12—C13—C14	178.7 (4)
C3—C4—C5—C6	−0.8 (6)	C11—C12—C13—C14	0.7 (6)
C4—C5—C6—Br3	178.9 (3)	C12—C13—C14—C15	0.2 (7)
C4—C5—C6—C1	−1.1 (6)	C13—C14—C15—C16	−0.9 (7)
N2—C7—C8—O1	−3.0 (7)	C14—C15—C16—C11	0.7 (7)
N2—C7—C8—C9	177.3 (4)		

### Hydrogen-bond geometry (Å, º)

**Table:**

*D*—H···*A*	*D*—H	H···*A*	*D*···*A*	*D*—H···*A*
O1—H1···N1	0.82	1.85	2.561 (4)	144
